# Two-year evaluation of Intermittent Preventive Treatment for Children (IPTc) combined with timely home treatment for malaria control in Ghana

**DOI:** 10.1186/1475-2875-10-127

**Published:** 2011-05-15

**Authors:** Collins K Ahorlu, Kwadwo A Koram, Atsu Seake-Kwawu, Mitchell G Weiss

**Affiliations:** 1 Noguchi Memorial Institute for Medical Research, University of Ghana, Box LG581, Legon, Ghana; 2 Keta District Health Management Team, Box KW198, Keta, Ghana; 3 Swiss Tropical and Public Health Institute, Soncinstrasse 57, CH-4002, University of Basel, Switzerland

## Abstract

**Background:**

Intermittent preventive treatment (IPT) has recently been accepted as an important component of the malaria control strategy. Intermittent preventive treatment for children (IPTc) combined with timely treatment of malaria related febrile illness at home to reduce parasite prevalence and malaria morbidity in children aged between six and 60 months in a coastal community in Ghana. This paper reports persistence of reduced parasitaemia two years into the intervention. The baseline and year-one-evaluation findings were published earlier.

**Objective:**

The main objective in the second year was to demonstrate whether the two interventions would further reduce parasite prevalence and malaria-related febrile illness in the study population.

**Methods:**

This was an intervention study designed to compare baseline and evaluation findings without a control group. The study combined home-based delivery of intermittent preventive treatment for children (IPTc) aged 6 - 60 months and home treatment of suspected febrile malaria-related illness within 24 hours. All children aged 6 - 60 months received home-based delivery of intermittent preventive treatment using amodiaquine + artesunate, delivered at home by community assistants every four months (6 times in 24 months). Malaria parasite prevalence surveys were conducted before the first and after the third and sixth IPTc to the children. The evaluation surveys were done four months after the third and sixth IPTc was given.

**Results:**

Parasite prevalence which reduced from 25% to 3.0% at year-one evaluation had reduced further from 3% to 1% at year-two-evaluation. At baseline, 13.8% of the children were febrile (axilary temperature of ≥37.5°C) compared to 2.2% at year-one-evaluation while 2.1% were febrile at year-two-evaluation.

**Conclusion:**

The year-two-evaluation result indicates that IPTc given three times in a year (every four months) combined with timely treatment of febrile malaria illness, is effective to reduce malaria parasite prevalence in children aged 6 to 60 months in the study community. This must give hope to malaria control programme managers in sub-Saharan Africa where the burden of the disease is most debilitating.

## Background

Malaria is estimated to cause between 300 and 500 million clinical cases with about 700,000 to 1.6 million deaths every year. About 94% of these deaths are believed to occur in sub-Saharan Africa [1,2]. In 2006 malaria accounted for 37.5% of all outpatient clinic visits, 36.0% of all admissions and about 33.4% of all deaths in children under five years of age in Ghana [3]. In line with WHO recommendation and the observed increasing treatment failure rate of chloroquine, the Ghana National Malaria Control Programme, (GNMCP) has adopted the use of artesunate and amodiaquine for the treatment of uncomplicated malaria in the country since January, 2005 [4,5].

Like elsewhere in Africa, malaria control in Ghana relies on early diagnosis and prompt treatment of suspected cases (fevers) and the home is where early recognition and, in most cases, prompt treatment is initiated [6-8]. IPTc delivered at home (community-based approach) was found to achieve a higher coverage compared to delivering at the clinic (facility-based approached) [9]. This is an indication that a lot more could be achieved through community involvement in malaria control activities.

Malaria control through prompt and effective treatment remains a challenge, especially in areas where access to early effective treatment is a problem. It is in these areas where IPT, especially IPTc could become an important component of the malaria control strategy, since it has been demonstrated that IPTi given with childhood vaccinations reduced incidence of first episode of malaria and also reduced severe anaemia by more than 50% during the first year of life [10-13]. It was previously shown that when given three times in a year this could reduce malaria parasite prevalence by over 80% [8].

### Rationale

The Ghana Health Service had aimed at treating 55% of under-five febrile malaria cases within 24 hours of symptom onset by 2007, as opposed to the Abuja target of 60% by 2005, but three clear years after the expiration date of 2007, this target is yet to be achieved. This study was, therefore, designed to assess the potential of a strategy of IPTc delivered at home and timely treatment of febrile illness to reduce parasite prevalence and malaria morbidity in the children-under 5 years as well as contribute to achieving the target of getting 55% of children under five into treatment within 24 hours. The baseline and year-one-evaluation results were published in 2009 [8]. This paper focuses on the year-two-evaluation, making reference to the earlier findings to better explain the current findings.

### Objectives

This paper is to further demonstrate the feasibility of controlling febrile malaria illness in the home through combined interventions of intermittent preventive treatment and timely febrile malaria illness management in children aged 6 - 60 months in southern Ghana [8].

## Methods

### Study site

The study site was discussed in detail in the earlier publication [8]. In brief, the study was conducted at Shime sub-district of the Keta district in the Volta region of Ghana, where malaria accounts for over 40% outpatients' clinic attendance in the District (District Annual Report, 2006). The study took place in an area with a population of 8,000. Community entry activities, involving meetings and durbars of chiefs and people in the study communities, were done at the onset of the project. After gaining consent from caretakers, children aged six to 60 months were enrolled into the study and no caretaker refused participation [8].

### Interventions

This was an intervention study designed to compare baseline and evaluation findings without a control group.

### Intermittent preventive treatment for children (IPTc)

IPTc was delivered every four months beginning in July 2007 (July, November 2007 and March 2008) and was evaluated in July, four months after the last round of IPTc and just before the fourth round of IPTc [8]. Just like the first year, IPTc was given three times (July, November 2008 and March 2009) and the evaluation took place four months after a total of six IPTc rounds in 24 months. Children received 10 mg/kg body weight of amodiaquine (AQ) and 4 mg/kg of artesunate (AS) daily (given as a single dose) over three days. All treatments were given under direct observation and children were observed for five minutes after drug administration to ensure that they retained the medication. Children who vomited within five minutes after taking the medicines received a repeated full dose. Children were followed up on day 1, 2 and 3 after treatment (the day of treatment was counted as Day 1) with adverse event questionnaire [8].

### Timely treatment of suspected febrile malaria in children

Any child with febrile illness suspected to have malaria by the caretaker was reported to the community assistants who then evaluated and treated those who met the protocol criteria for suspected febrile malaria illness, which was a child with axillary temperature of ≥ 37.5°C; whose caretaker reported suspected febrile malaria together with one or more of the following; headache, hot body, weakness, loss of appetite, among others, with no other overtly observed or reported condition was treated with the full three-day course of artesunate and amodiaquine [8].

### Questionnaire survey

During IPTc rounds and prevalence surveys, a simple questionnaire was administered to caretakers to record febrile illnesses in the seven days prior to those events. That is, caretakers whose children received IPTc at any round and those who participated in the prevalence surveys responded to the questions. Questions asked included: Has your child been well in the past seven days, including now? If no, what is/was wrong with him/her? What was done for the child when you found out that he/she was not well? During the prevalence surveys, however, additional questions were asked about bed net usage.

### Sample size

This was an implementation research which aimed at enrolling every child aged six to 60 months living in the study area into the study with the consent of their caretakers, mostly parents. However, for the prevalence survey, a sample size of 174 was needed to determine malaria parasite prevalence estimated at 20% in the study population [14]. At the evaluations however, all available participants at the time were examined. This led to the examination of over 85% of children enrolled into the study at both year-one and year-two evaluations. Data were analysed to compare results from year-one and year-two evaluation with reference to the baseline results. All quantitative analyses were done using Epi Info version 3.4.1.

### Laboratory methods

A diagnosis of malaria infection was made with light microscopy of thick and thin blood smears. The thick and thin blood smears were stained with 3% Giemsa for 30 minutes. Parasite density was determined by counting the number of asexual parasites per 200 white blood cells, and calculated per μL assuming a white blood cell count of 8,000 cells per μL. Sexual parasite count was done per 1,000 white blood cells. A smear was declared negative if examination of 100 thick-film fields did not reveal the presence of asexual parasites. Quality control was done by allowing a second microscopist to read a random sample of 10% of all slides to confirm the absence or presence of parasites. Haemoglobin concentration was determined from finger pricks using a portable automated Hemocue^® ^photometer (Leo Diagnostics, Sweden) at the field site [8].

### Ethical review

The Institutional Review Board of the Noguchi Memorial Institute for Medical Research, University of Ghana and the Ethical Review Committee of WHO reviewed and approved the study. Each mother/caretaker was informed of the objectives, methods, anticipated benefits and potential hazards of the study. Caretakers were also informed that they were at liberty to withdraw their children from the study at any time without penalty. They were assured that all information collected during this study would be kept confidential and that in any resulting publication it would not be possible to link the data to individuals in the study [8].

## Results

This paper presents the two-year-evaluation of combined IPTc and timely treatment febrile illnesses (suspected clinical malaria). Most of the children received IPTc six times in 24 months (on average 420 children were seen on each round, ranging between 413 and 433).

### Parasite prevalence

Malaria parasite prevalence at year-one evaluation was 3.0% (10 out of 357), which further dropped significantly to about 1.0% (3 out of 337) at year-two-evaluation, a significant reduction of over 60.0%, p < 0.001, Fisher exact test). This showed a point parasite prevalence reduction of over 90.0% in 24 months (25.3% at baseline to 1.0% at year-two-evaluation). There was a significant relationship between febrile status and parasitaemia at year-one-evaluation (p < 0.001, Fisher exact test) but this was not the case at year-two evaluation. Two out of the three parasite positive cases receive IPTc four times and the remaining one received it twice. All the positive infections were *Plasmodium falciparum*, the dominant species in Ghana. Other clinical and parasitological findings are presented in Table 1.

**Table 1:** Baseline, Year 1 and 2 Evaluation Characteristics, Clinical and Parasitological findings

Variables	Baseline (N = 174)	Evaluation Year 1 (N = 357)	Evaluation Year 2 (N = 365)
Mean age in months (±std.)	30.5 (16.5) months	37.7 (15.8) months	38.19 (15.7) months
Sex (% of female)	52.3	47.0	50.1
Weight: Mean (±std.)	12.3 (3.5)	13.8 (3.5)	12.2 (3.3)
Haemoglobin < 10 g/dl (%) ***	48 (27.6)	60 (16.8)	53 (15.7)
Number (%) febrile ≥ 37.5°C **	24 (13.8)	8 (2.2)	8 (2.1)
Parasite positive number (%) **	44 (25.3%)	10 (3.0)	3 (1.0)

±std. = standard deviation

** P = 0.000 and *** P = 0.003 (trend analysis)

### Anaemia

In this study, anaemia was defined as haemoglobin <10 g/dl and trend analysis showed a significant reduction from baseline through to year-two evaluation (p = 0.003). Comparing the baseline level of 27.6% to year-two evaluation level of 15.7% showed about 43.1% reduction in anaemia cases in 24 months (Table 1). There was a significant relationship between febrile status and anaemia at year-one evaluation (P = 0.01 (Fisher exact test)), but this was not the case at year-two-evaluation.

During the prevalence surveys (baseline and evaluations) a number of febrile illnesses were reported for the children within seven days prior to data collection (Table 2). A trend analysis showed a significant reduction (p < 0.001). The point prevalence of fever cases showed a reduction of over 85.0% when the baseline level of 42% is compared to the year-two evaluation level of 4.7%.

**Table 2:** The distribution of febrile illnesses reported for children within the past seven days prior to the baseline and evaluation prevalence surveys. *

Variables	Baseline (N = 174)	Evaluation Year1 (N = 357)	Evaluation Year 2 (N = 337)
Chills and Rigors **	18 (10.3)	13 (3.6)	4 (1.2)
Convulsions	4 (2.3)	0	0
Diarrhoea **	32 (18.4)	15 (4.2)	2 (1.2)
Fever/Malaria/Asra **	73 (42.0)	27 (7.6)	16 (4.7)
Headache **	58 (33.3)	18 (5.0)	6 (1.8)
Others (Cough, Rashes, Stomach ache etc.) ***	20 (11.5)	8 (2.2)	16 (4.7)
Vomiting **	20 (11.5)	11 (3.1)	2 (0.6)

* Sorted in column 1 in alphabetical order.

** P < 0.001 and *** P = 0.011 (trend analysis)

### Intermittent preventive treatment for children (IPTc)

IPTc was delivered to children aged 6 to 60 months in the study community six times (July and November 2007; March, July and November 2008 and March 2009). There have been marginal increases in the number of children at each subsequent treatment round until the fourth, where it dropped marginally from 433 (IPTc3) to 415 (IPTc4), and then increased to 419 (IPTc5) and 421 (IPTc6). The decrease was largely due to some children attaining the age of more than 60 months and were dropped out of the study. At the time of year-two-evaluation, majority of the children have received all six IPTc doses. A few were lost at various IPTc rounds due mainly to either the movement of these children out of the study community at one point or the other, or the entry points of participants, since enrolment was prospective and any child who attained the age of six month at any given time was enrolled. However, the number of dropouts at each round was too small for any meaningful comparative analysis to be done on them. During each IPTc round, a number of febrile illnesses were reported by caretakers within the past seven days prior to treatment and trend analysis showed significant reductions in reported fever as the number of IPTc rounds increases (Table 3).

**Table 3:** The distribution of febrile illnesses reported for children within the past seven days prior to each drug administration. *

Febrile illness reported in the past seven days prior to drug administration	IPTC1 (N = 413)	IPTC2 (N = 420)	IPTC3 (N = 433)	IPTC4 (N = 415)	IPTC5 (N = 419)	IPTC6 (N = 421)
	
	Number (%)	Number (%)	Number (%)	Number (%)	Number (%)	Number (%)
Fever/Malaria/Asra†**	64 (15.50)	43 (10.20)	11 (2.50)	8 (1.93)	9 (2.15)	7 (1.66)
Headache**	49 (11.90)	39 (9.30)	5 (1.20)	8 (1.93)	11 (2.62)	7 (1.66)
Vomiting**	25 (6.10)	11 (2.60)	1 (0.20)	1 (0.24)	5 (1.19)	2 (0.47)
Chills and Rigors***	17 (4.10)	10 (2.40)	7 (1.60)	5 (1.20)	6 (1.43)	5 (1.19)
Others (Cough, Rashes, Stomach ache etc.	7 (1.70)	5 (1.20)	9 (2.10)	12 (2.89)	8 (1.91)	10 (2.37)
Convulsions	5 (1.20)	0	0	0	0	0
Diarrhoea	5 (1.20)	1 (0.20)	3 (0.70)	2 (0.48)	2 (0.48)	1 (0.24)

*Sorted in column two in descending order to draw attention to the higher figures reported in that column

** P = 0.000 and *** P = 0.002 (trend analysis)

*† 'Asra' *is a local term used collectively to represent fever and/or malaria in the study community

A number of febrile malaria-related illnesses were treated by community assistants in between IPTc rounds and trend analysis showed no significant reduction in these reported cases, when analysed from the first round. However, interviews with caretakers revealed that they were sceptical about the ability of community assistants to manage the febrile illnesses of their children until after the second rounds of IPTc, so it was decided to perform a second trend analysis starting from the second round of IPTc, and this showed a significant reduction in the reported febrile malaria/fever (<0.001) and headache (0.021) reported as the IPTc rounds increased but not for chills and rigors, and vomiting (Table 4).

**Table 4:** Febrile illnesses treated by community assistants in between IPTC rounds**

Febrile illness treated between TPTC rounds	IPTC1-IPTC2 (N = 413)	IPTC2-IPTC3 (N = 420)	IPTC3-IPTC4 (N = 433)	IPTC4-IPTC5 (N = 415)	IPTC5-IPTC6 (N = 419)
	
	Number (%)	Number (%)	Number (%)	Number (%)	Number (%)
Fever/Malaria/*Asra*	5 (1.21)	39 (9.28)	17 (3.93)	13 (3.13)	10 (2.39)
Headache	6 (1.45)	24 (5.71)	17 (3.93)	14 (3.37)	11 (2.62)
Chills and Rigors	4 (0.98)	12 (2.86)	9 (2.08)	11 (2.65)	7 (1.67)
Vomiting	0	4 (0.95)	3 (0.70)	8 (1.93)	4 (0.96)

**Sorted in column three in descending order to draw attention to the higher figures reported in that column

There was virtually no serious adverse event reported after drug administrations. However, some caretakers reported that their children were weak for about two to four hours after taking the medicines and these were; five (1.21%) IPTc1, four (0.95%) IPTc2, seven (1.65%) IPTc3, four (0.93%) IPTc4, five (1.13%) IPTc5 and 5 (1.11%) IPTc6. None of these cases warranted any medical intervention. A few caretakers also reported that their children did eat very well after taking the drugs but this could not be independently verified.

As this was at year-one evaluation, no caretaker had lost a child in 12 months preceding the year-two evaluation, indicating that, no caretaker had lost a child in the twenty-four months of project implementation, except two infants who died at birth and this was attributed to the absence of a midwife at the clinic at the time of their birth.

### Bed net usage

As expected, over 98% of the caretakers were mothers of the children. Caretakers (98.0% at baseline and 100% at both year-one and year-two evaluations) reported that they and their families slept under bed nets the previous night. Comparatively, insecticide-treated net usage has increased marginally from 60.0% at year-one evaluation to 63.0% at year-two evaluation. However, compared to baseline (38.5%), there was a significant increase of 63.6% in the use of treated bed net in 24 months.

## Discussion

This paper presents the two-year-evaluation of intermittent preventive treatment for children (IPTc) combined with timely treatment at home for malaria control, targeting children aged 6 - 60 months old. The main finding at year-two-evaluation was a further reduction of about 66.6% (from 3.0% at year-one-evaluation to 1.0% at year-two-evaluation) in malaria prevalence in the study population compared to about 88.0% reduction reported between baseline and year-one-evaluation (25.0% at baseline and 3.0% at year-one-evaluation) [8]. Compared to baseline (25.0%), year-two-evaluation shows a reduction of over 90.0% in parasite level in the study population. Several studies in IPT intervention measured clinical incidence rather than prevalence and found between 20% and 86.0% reduction with strong variations depending on transmission duration and intensity, target population and intervals between treatments [9-12,15-18].

Anaemia in the children, defined as haemoglobin <10g/dl, further improved by 27.4% (from 16.8% at year-one evaluation to 12.2% at year-two evaluation). Compared to baseline, this was over 65.0% reduction in all cause anaemia prevalence in the study population. This compares well with a recent randomized trial in Tanzania, which showed that IPT given to infants at the time of childhood immunization reduced the incidence of the first episode of malaria and anaemia by more than 50.0% during the first year of life [11,12]. However, the reduction in anaemia prevalence at year-two evaluation compared to year-one evaluation was not as dramatic as it was at year-one-evaluation compared to baseline. This could be attributable to the low level of parasitaemia observed after the year-one evaluation. Similar observation was also reported from The Gambia [19]. Thus, at low prevalence, malaria may be contributing less to anaemia in children aged 6-60 months.

Malaria-related morbidity in the study population, expressed by the presence of fever and other malaria-related signs and symptoms reported, has reduced as captured either during prevalence surveys or IPTc administration. Timely treatment of febrile malaria cases in the community did not follow any pattern. However, it could be argued that the community assistants who were on hand to deliver effective treatment in a timely fashion have contributed to the marked reduction of parastaemia seen during evaluation surveys. A community randomized trial showed a slightly higher coverage in the community-based delivery arm compared to facility-based arm [9]. This should encourage malaria control programmes to have confidence in community assistants to deliver timely treatment and possibly IPTc to children at community levels, once they are well trained by the programme, coupled with reference treatment card for easy and quick referencing when in doubt [8].

Findings reported here present a challenge to the existing practice, especially in most sub-Saharan African countries where malaria diagnosis is mostly based on presumption without confirmation. As community interventions or access to treatment increases, this may lead to fewer malaria infections, which may pose the danger of over diagnosis and treatment with expensive drugs for people who do not need them [8]. Control programmes should therefore invest in rapid diagnostic test kits where microscopy is not possible. As reported by Zikusooka *et al *[20], this may lead to cost savings because artemisinin-based anti-malarial drugs are expensive. Goodman *et al *[21] also make this point, as rational use of anti-malarials will reduce the potential for emergence of resistance.

The use of IPTc combined with timely home treatment to control malaria was found to reduce malaria prevalence in children aged six to 60 months [8]. Although this study cannot determine the contribution of IPTc or timely home treatment to the protection offered to the children because the two interventions were delivered concurrently, the two together in this study offered a major protection against malaria in children, reducing prevalence from 25.0% at baseline to 1.0% at year-two evaluation (twenty-four months of implementation). The question then is, at what point should the provision of IPTc stop? What techniques or methods could be used to mop-up the few parasites that may continue to sustain transmission, because the vector mosquito is highly efficient in malaria transmission?

At this point, it may be recommended to reduce the frequency of treatment from every four months to every six months or providing treatment at the beginning of each peak season (two peak seasons in southern Ghana). Either way, the timing must be related to the major peak season in the study area. At certain point it should be necessary to treat the general population at least once in a year to reduce the circulating parasites in the population such that mosquitoes will have very little or no parasite to transmit. Findings reported in this paper indicates that, with consistent efforts, malaria could be put under control such that it may no longer be a major public health problem, especially in sub-Sahara Africa where it is a threat to millions of people. Also, the study shows that it is possible to train community assistants to deliver IPTc together with home management of malaria on timely basis with very little supervision. However, additional training must be provided to enable community assistance to use RDT in the community so as to move away from presumptive treatment as malaria incidence continues to go down in communities.

### Study limitations

One major limitation of the study was that rapid diagnostic test (RDT) was not used to confirm the presence of parasite before timely treatment was provided as a result of the ethical consideration not to allow field assistants to take blood from the children. Another weakness of the study is that there was no control group to compare the result so it was not possible to attribute all the reduction in parasite prevalence to the interventions. Also, the contribution of each of the intervention to the reduction of parasite prevalence in the study population was not measured. All these must be taken into consideration when designing s similar study in future.

## Competing interests

The authors declare that they have no competing interests.

## Authors' contributions

CKA was involved in the conception, design and implementation, data analysis and writing of this paper. KAK was involved in the design and writing of this paper. AS-K was involved in the implementation and writing of this paper. MGW was involved in the conception, design and writing of this paper. All the authors have read and approved the final version of the manuscript.
